# Posterior reversible encephalopathy syndrome in a patient with Richter's syndrome on combination DA-R-EPOCH chemotherapy regimen: a case report

**DOI:** 10.1186/s13256-021-02780-y

**Published:** 2021-04-12

**Authors:** Goar Egoryan, Ricardo Murguia-Fuentes, Mohamed Agab, Nagwa Abou-Ghanem, Maria Adriana Yanez-Bello, Bimatshu Pyakuryal, Daniela Patricia Trelles-Garcia, Rami Ibrahim, Dorota Filipiuk, Adi Gidron, Guillermo Rodriguez-Nava

**Affiliations:** 1 Department of Internal Medicine, AMITA Health Saint Francis Hospital, 355 Ridge Ave, Evanston, IL 60202 USA; 2 Department of Neurology, Louisiana State University Health Sciences Center, Shreveport, LA USA; 3 Department of Radiology, AMITA Health Saint Francis Hospital, Evanston, IL USA; 4 Department of Pathology, AMITA Health Saint Francis Hospital, Evanston, IL USA; 5 Department of Hematology-Oncology, AMITA Health Saint Francis Hospital, Evanston, IL USA

**Keywords:** Posterior reversible encephalopathy syndrome, Chronic lymphocytic leukemia, Diffuse large B-cell lymphoma, Richter syndrome, DA-R-EPOCH, R-EPOCH

## Abstract

**Background:**

Posterior reversible encephalopathy syndrome (PRES) is a clinical-radiologic entity characterized by headaches, altered mental status, seizures, visual loss, and a characteristic imaging pattern in brain magnetic resonance images. The exact etiology and pathogenesis of this condition are not yet fully elucidated.

**Case presentation:**

A 72-year-old White man presented with 2 weeks of low-grade fever and chills, night sweats, fatigue, dysphagia, and new-onset rapidly increasing cervical lymphadenopathy. He had a history of chronic lymphocytic leukemia with transformation to diffuse large B-cell lymphoma for which he was started on dose-adjusted rituximab, etoposide, prednisone vincristine, cyclophosphamide, and doxorubicin (DA-R-EPOCH). Shortly after treatment initiation, the patient developed severe airway obstruction due to cervical lymphadenopathy that required emergency intubation. A few days later, the cervical lymphadenopathy and the status of the airway improved, and sedation was consequently weaned off to plan for extubation. However, the patient did not recover consciousness and developed generalized refractory seizures. Brain magnetic resonance imaging revealed edema in the cortical gray and subcortical white matter of the bilateral occipital and inferior temporal lobes, consistent with PRES.

**Conclusions:**

Posterior reversible encephalopathy syndrome refers to a neurological disorder and imaging entity characterized by subcortical vasogenic edema in patients who develop acute neurological signs and symptoms of a usually reversible nature in different settings, including chemotherapy. Despite its name, PRES is not always fully reversible, and permanent sequelae can persist in some patients. Clinicians should be aware of the possible association between chemotherapy and PRES to ensure early recognition and timely treatment.

## Background

The conversion to aggressive lymphoma in patients with chronic lymphocytic leukemia (CLL), or Richter syndrome, is a concerning complication that carries a very poor prognosis, with a usual median survival of between 8 and 21 months [1]. The combination chemotherapy regimen of etoposide, prednisone, vincristine, cyclophosphamide, doxorubicin, and the relatively recent addition of rituximab, also known as R-EPOCH (or DA-R-EPOCH for dose-adjusted regimen), has improved the management and outcome of these hematological malignancies [2]. Despite some existing data, at the present time, no single chemotherapeutic agent has been established to have a consistent association with the development of posterior reversible encephalopathy syndrome (PRES), although among the chemotherapy drugs, cisplatin, cytarabine, adriamycin, cyclophosphamide, and the vinca alkaloids have been occasionally implicated with this syndrome [11]. Some published data has also linked the DA-EPOCH combination chemotherapy regimen with PRES. Despite its rarity, this condition should be readily recognized and appropriately managed.

## Case presentation

**Fig. 1:**
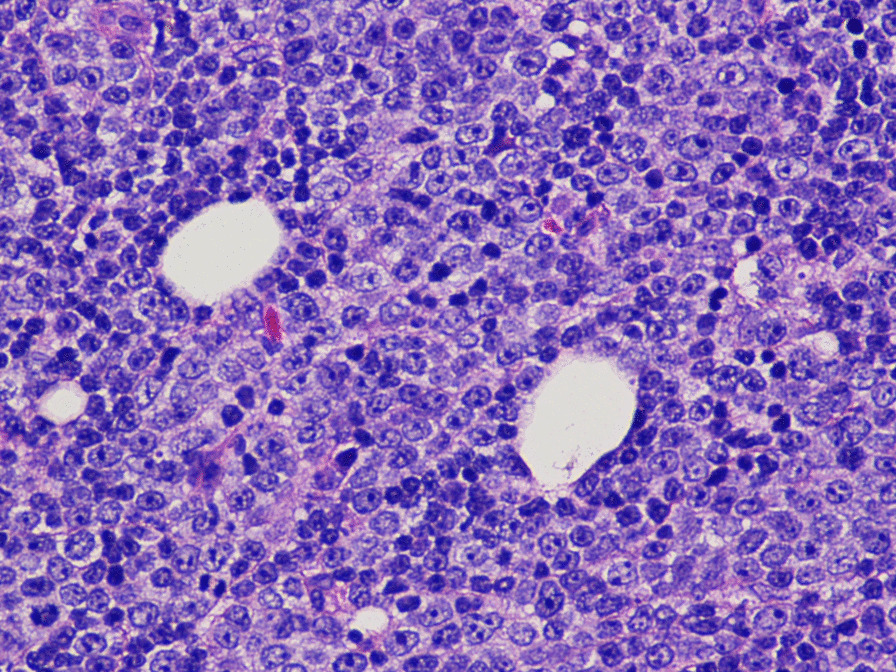
Lymph node biopsy showing Richter syndrome. The lymph node architecture was effaced by a diffuse proliferation of large lymphoid cells with features of immunoblasts. A starry sky pattern and frequent mitoses were also noted. The large cells were intermixed with a numerically smaller population of small, mature-appearing lymphocytes. The large cells were positive for CD20, CD79a, BCL6, and MUM1 and negative for CD10 and CD3 by immunohistochemistry. The Ki-67 proliferation index was approximately 80%, and in situ hybridization for Epstein-Barr virus was negative. According to Hans' algorithm, these findings are consistent with diffuse large B-cell lymphoma, activated B-cell subtype, arising in a background of chronic lymphocytic leukemia/small lymphocytic lymphoma

A 72-year-old White man with an 8-year history of CLL previously treated with four cycles of fludarabine, cyclophosphamide, rituximab in 2012, and ibrutinib since 2014, presented to the oncology clinic with 2 weeks of low-grade fever and chills, night sweats, fatigue, dysphagia, and new-onset rapidly increasing cervical lymphadenopathy. The interval workup revealed worsening of lymphocytosis (61% at presentation vs. 40.2% at 6 months before the presentation) and thrombocytopenia (42 vs. 92 K/mm^3^). A positron emission tomography scan showed bulky confluent hypermetabolic adenopathy throughout all bilateral neck, chest, abdomen, and pelvis portions. The patient was subsequently admitted to the hospital with suspected CLL progression and possible transformation to a more aggressive type. Lymph node biopsy revealed that high-grade diffuse large B-cell lymphoma (DLBCL) had transformed from CLL (Richter syndrome; Fig. 1). The patient was started on DA-R-EPOCH chemotherapy. However, the patient's course was complicated by tumor lysis syndrome, acute kidney injury, and neutropenic fever with *Pseudomonas aeruginosa* bacteremia. Therapy with broad-spectrum antibacterials and antifungals and with granulocyte colony-stimulating factor (G-CSF) was initiated.

**Fig. 2:**
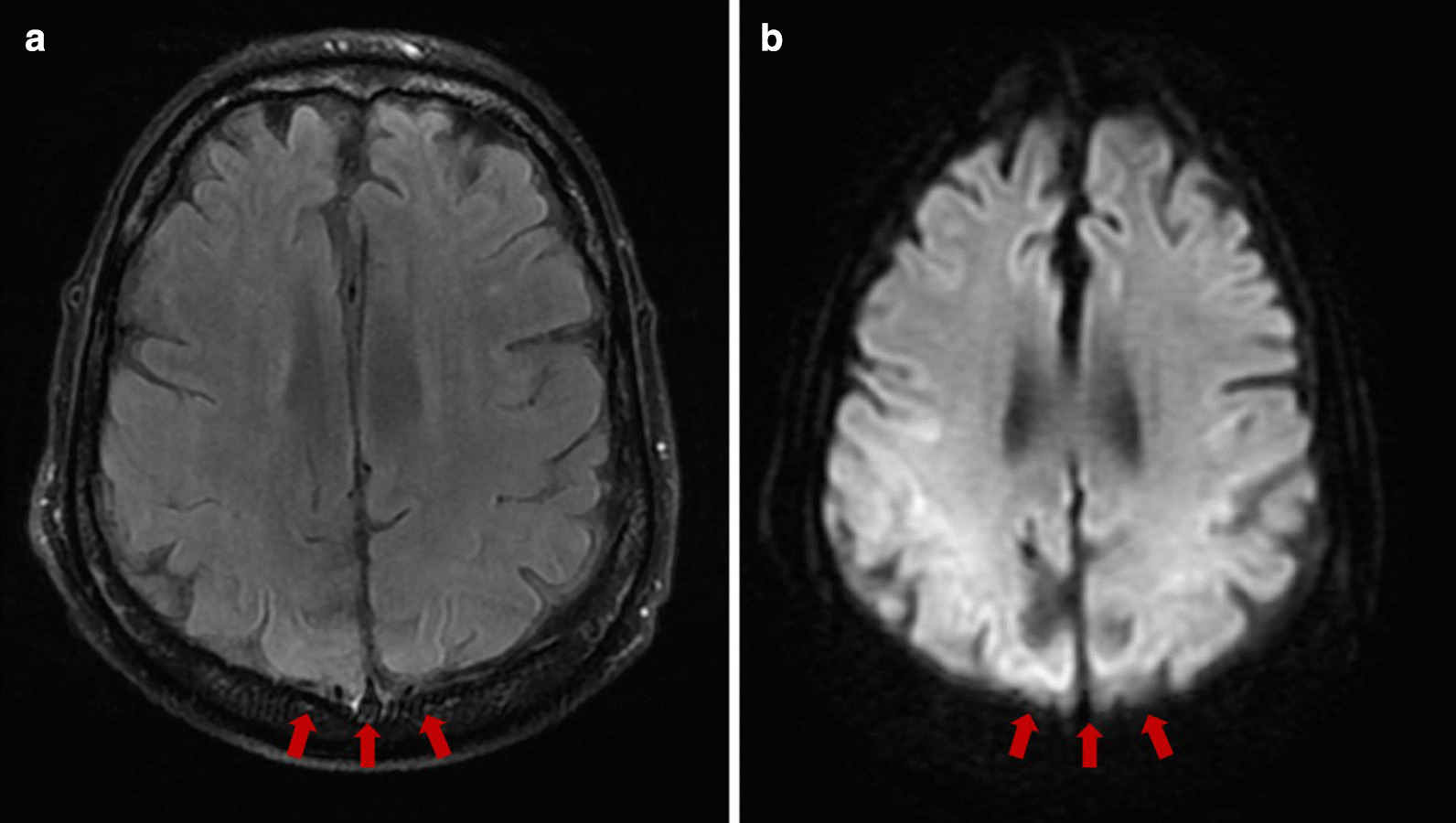
Brain magnetic resonance imaging (MRI). **a** T2 fluid-attenuated inversion recovery (FLAIR) sequence showed mild hyperintensity of the occipital and inferior temporal lobes bilaterally (red arrows). **b** Diffusion-weighted imaging demonstrated restricted diffusion in the cortical gray and subcortical white matter of the same regions (red arrows) consistent with edema in the cortical gray and subcortical white matter

Despite an improvement in cervical lymphadenopathy, on day 9 of hospitalization, the patient developed severe airway obstruction and immediately underwent emergency intubation for airway protection, following which he was transferred to the intensive care unit (ICU). Magnetic resonance imaging (MRI) of the neck demonstrated significant neck soft-tissue edema, for which he received high doses of hydrocortisone, but with no improvement. On day 10, the patient remained unresponsive after sedation was weaned off, and he subsequently developed two episodes of generalized seizures. Computed tomography of the head showed no acute abnormalities. An electroencephalogram did not demonstrate any signs of focal or generalized seizure activity. Brain MRI revealed edema in the cortical gray and subcortical white matter of the bilateral occipital and inferior temporal lobes, consistent with posterior reversible encephalopathy syndrome (Fig. 2). The patient experienced recurrent seizure episodes refractory to lorazepam, levetiracetam, valproate, but responsive to midazolam and propofol. Of note, additional blood cultures became positive for *Klebsiella oxytoca;* thus, tigecycline was added to the antibiotic regimen. However, considering the overall grim prognosis, the decision was made, in conjunction with the family, not to escalate care. Unfortunately, the patient's condition deteriorated further, resulting in cardiac arrest. We concluded that the nature of PRES, in this case, was multifactorial and related to the recently initiated combination chemotherapy, with acute kidney injury and sepsis being essential additional risk factors.

## Discussion and conclusion

Posterior reversible encephalopathy syndrome refers to a neurological disorder and imaging entity characterized by subcortical vasogenic edema in patients who develop acute neurological signs and symptoms of a usually reversible nature in different settings [3, 4]. This disorder is characterized by acute or subacute onset of symptoms, including headache, impaired visual acuity, visual field deficits, changes in consciousness, confusion, and focal deficits [3]. Seizures can occur in about two-thirds of the patients [5]. While epidemiological data suggest an increased prevalence in middle-aged female patients, it can affect all age groups, including older adults such as the patient presented in this case. Accurate epidemiological data are controversial, partly due to the challenges of confirming the diagnosis and subsequent under-identification of the syndrome [6, 7].

While not a radiological diagnosis by itself, PRES requires imaging support to complement the clinical context. Imaging findings include vasogenic edema, watershed distribution, and a parieto-occipital pattern with areas of T2 signal abnormality on MR images [3, 4]. However, the involvement of frontal and temporal lobes is also common. MRI diffusion-weighted imaging can be useful to distinguish between cytotoxic edema (hyperintensity on imaging) and vasogenic edema (iso- or hypointensity on imaging) [12]. It is relevant to mention that fluid-attenuated inversion recovery (FLAIR) has a higher sensitivity as an imaging modality for diagnosing PRES [8].

Although the exact mechanism is unknown, it is believed that cytotoxic drugs disrupt the function of the blood–brain barrier and generate vasogenic edema through endothelial dysfunction and failure of cerebral autoregulation [9]. As chemotherapy use keeps increasing, there is a higher likelihood that PRES may be diagnosed more frequently in the future. When medical conditions such as acute hypertension, renal dysfunction, or electrolyte imbalances are present, the risk of developing PRES increases, as illustrated in our case [10, 11].

Currently, no single chemotherapeutic agent has been consistently associated with PRES. Chemotherapy drugs that have been occasionally implicated include cisplatin, cytarabine, adriamycin, cyclophosphamide, and the vinca alkaloids [11]. Floeter et al. reported that the DA-EPOCH regimen caused PRES in three of 44 patients (7%) in a retrospective study at their institution [12]. Several risk factors, including pre-existing central nervous system (CNS) insult, changes in the fluid status, electrolyte abnormalities, and hypertension, were considered in their analysis. However, their study included a small number of patients, and no reported cases involved the addition of rituximab to the therapeutic regimen [12]. These limiting factors leave undetermined if the addition of rituximab makes a difference in the likelihood of PRES occurrence.

PRES management is mostly symptomatic, with blood pressure and seizure management being essential components [4]. When secondary to cytotoxic medications, such as chemotherapy, it is still controversial whether tapering off or suddenly discontinuing chemotherapy is beneficial. One study compared three interventions after tacrolimus-induced PRES in pediatric patients: one group continued taking the same dose of tacrolimus, the second group discontinued the treatment altogether, and the third group was switched to another agent. No differences in mortality were identified [13]. Further studies are needed to identify if a similar scenario occurs in patients receiving chemotherapy.

Despite its name, PRES is not always fully reversible, and long-lasting sequelae can persist in 10–20% of patients [14]. The prognosis is usually favorable, and most of the patients fully recover. In our patient, mortality was likely driven by the development of bacteremia, septic shock, and progressive renal failure rather than a neurological etiology.

To our knowledge, this report represents the first description of DA R-EPOCH combination chemotherapy associated with PRES. In addition to the chemotherapy, the patient also had progressively worsening acute kidney injury and sepsis as risk factors, which may have increased the likelihood of developing this neurological condition and further deterioration leading to death. More research is required to identify the incidence and the strength of association of PRES with various chemotherapy regimens, especially DA R-EPOCH, and to delineate the populations at risk. Clinicians should be aware of the possible association between chemotherapy and PRES to ensure early recognition and timely treatment.

## Data Availability

The data used to support the findings of this study are available from the corresponding author on request, except for the patient's personal health information due to Health Insurance Portability and Accountability regulations.

## Abbreviations

CLL: Chronic lymphocytic leukemia
CNS: Central nervous system
DA-R-EPOCH: Dose-adjusted rituximab, etoposide, prednisone, vincristine, cyclophosphamide, and doxorubicin
DLBCL: Diffuse large B-cell lymphoma
FLAIR: Fluid-attenuated inversion recovery
G-CSF: Granulocyte colony-stimulating factor
ICU: Intensive care unit
MRI: Magnetic resonance imaging
PRES: Posterior reversible encephalopathy syndrome
